# Enhanced *in Vivo* Delivery of 5-Fluorouracil by Ethosomal Gels in Rabbit Ear Hypertrophic Scar Model

**DOI:** 10.3390/ijms151222786

**Published:** 2014-12-09

**Authors:** Yan Wo, Zheng Zhang, Yixin Zhang, Zhen Zhang, Kan Wang, Xiaohui Mao, Weijie Su, Ke Li, Daxiang Cui, Jun Chen

**Affiliations:** 1 Department of Human Anatomy, Histology and Embryology, School of Medicine, Shanghai Jiao Tong University, Shanghai 200025,China; E-Mail: woyansh@gmail.com; 2 Department of Plastic and Reconstructive Surgery, Shanghai Ninth People’s Hospital, School of Medicine, Shanghai Jiao Tong University, Shanghai 200011, China; E-Mails: zhangzheng958@gmail.com (Z.Z.); rosiewudi@163.com (W.S.); 18817821624@163.com (K.L.); 3 Department of Dermatology, Shanghai Ninth People’s Hospital, School of Medicine, Shanghai Jiao Tong University, Shanghai 200011, China; E-Mail: zz6503@126.com; 4 National Key Laboratory of Nano/Micro Fabrication Technology, Key Laboratory for Thin Film and Microfabrication of Ministry of Education, Institute of Micro-Nano Science and Technology, Shanghai Jiao Tong University, Shanghai 200240, China; E-Mails: wk_xa@163.com (K.W.); dxcui@sjtu.edu.cn (D.C.); 5 Department of Otolaryngology-Head and Neck Surgery, the Third People’s Hospital, School of Medicine, Shanghai Jiao Tong University, Shanghai 201900, China; E-Mail: mxh578694@163.com; 6 Department of Orthopaedic Sports Medicine, Huashan Hospital, Fudan University, Shanghai 200040, China; E-Mail: biochenjun@fudan.edu.cn

**Keywords:** drug delivery, ethosome, 5-fluorouracil, gel, hypertrophic scars penetration

## Abstract

Applying Ethosomal Gels (EGs) in transdermal drug delivery systems has evoked considerable interest because of their good water-solubility and biocompatibility. However, there has not been an explicit description of applying EGs as a vehicle for hypertrophic scars treatment. Here, a novel transdermal EGs loaded with 5-fluorouracil (5-FU EGs) was successfully prepared and characterized. The stability assay* in vitro* revealed that 5-FU EGs stored for a period of 30 days at 4 ± 1 °C had a better size stability than that at 25 ± 1 °C. Furthermore, using confocal laser scanning microscopy, EGs labeled with Rhodamine 6 G penetrated into the deep dermis of the hypertrophic scar within 24 h in the rabbit ear hypertrophic model suggested that the EGs were an optional delivery carrier through scar tissues. In addition, the value of the Scar Elevation Index (SEI) of 5-FU EGs group in the rabbit ear scar model was lower than that of 5-FU Phosphate Buffered Saline gel and Control groups. To conclude, these results suggest that EGs delivery system loaded 5-fluorouracil is a perfect candidate drug for hypertrophic scars therapy in future.

## 1. Introduction

Compared to normal skin, hypertrophic scars (HS) have different tissue composition that lacks mature corneocytes, and this leads to stratum corneum barrier dysfunction [1,2]. Thus, drug administration for HS tissue therapy is more difficult due to its fewer hair follicles, skin glands, dermal papilla, and other ancillary structures [3]. 5-fluorouracil (5-FU) is widely recommended in HS treatment because of its antiscarring activity [4]. Currently, the administration route is mainly intralesional injection. A disadvantage of this treatment includes severe pain caused by the injections (despite the use of anesthesia). Therefore, to develop a novel drugs administration to better penetrate HS tissues has become an increasing area of interest for research in recent years. In the past several decades, various approaches have been utilized to improve transdermal and topical drug delivery. Topical delivery of drugs by lipid carriers, such as ethosomes, has attracted considerable attention [5,6,7]. An ethosome is a novel lipid carrier, recently developed by Touitou* et al.*, that exhibits enhanced skin delivery of drugs and with low cytotoxicity* in vitro* and* in vivo* studies [8,9,10,11,12]. Our previous research found that the penetration of ethosomal suspensions had higher penetration rates in hypertrophic scars compared to hydroethanolic solution. Using high performance liquid chromatography, these results revealed that nanodiameter-ethosomal suspensions encapsulated with 5-fluorouracil have an ability to obtain retention effects* in vitro* [13,14,15]. However, ethosomal suspensions, due to their liquid nature, could not continue to be a topical drug administration, which will create problems in the future in applying them to hypertrophic scars.

Gel formulation is defined as a substantially diluted cross-linked system, which exhibits no flow when in the steady-state [16]. It is a dispersion of molecules of a liquid within a solid created by the cross-linking of each other to constitute a three-dimensional cross-linked network. In this way, gel formulation has properties between solid phase and liquid phase. Recently, gel formulation has become one of the most relevant routes for treating skin diseases efficaciously [17]. It is of practical significance using percutaneous drug delivery and transdermal absorption in clinics. Therefore, Ethosomal Gels’ (EGs’) formulation is a suitable drug carrier to facilitate the painless delivery of drugs into scar tissue. In this study, EGs were prepared as a delivery system for loading 5-FU at first. Then, their therapeutic effects for hypertrophic scar tissues were further evaluated by the rabbit ear hypertrophic scar model.

## 2. Results and Discussion

### 2.1. Preparation and Characterization of 5-Fluorouracil Ethosomal Gels (5-FU EG)

Since 2001, 5-FU was confirmed to inhibit fibroblasts proliferation in scar tissue [18]. It is widely applied in clinical settings as an aid in the medical conservative treatment of scar [19,20]. However, 5-FU is still given as a topical administration that produces marked side effects such as intense pain, ulceration, tissue atrophy, purpura, and pigmentation [21,22]. Ethosomal vesicles which are soft and malleable can permeate the immature corneocytes into hypertrophic scar as well as the hydrophobic ceramide composition of the hypertrophic scar stratum corneum lipids is decreased which allows easier penetration of drugs into scar tissues [15,23]. In addition, a change in the formulations to transdermal delivery avoids the side effects caused by topical administration and is well accepted by patients. Choosing the suitable substrate is important for treatment of scar.

**Figure 1:**
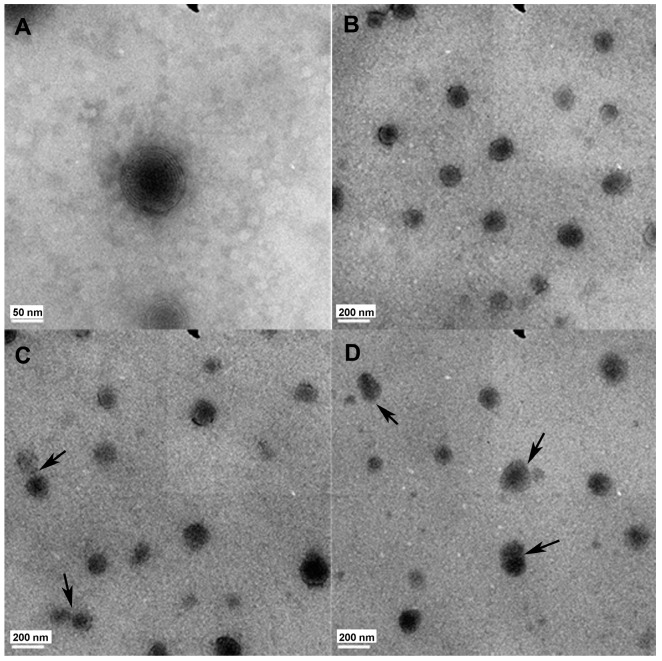
Transmission electron (TEM) images of ethosomal gel carried with 5-Fluorouracil (5-FU). (**A**,**B**) Freshly prepared 5-FU Ethosomal Gels (EGs); (**C**) 5-FU EGs stored at 4 ± 1 °C for 30 days; (**D**) 5-FU EGs stored at 25 ± 1 °C for 30 days; Black arrow indicated these vesicles of ethosomal gel.

**Figure 2:**
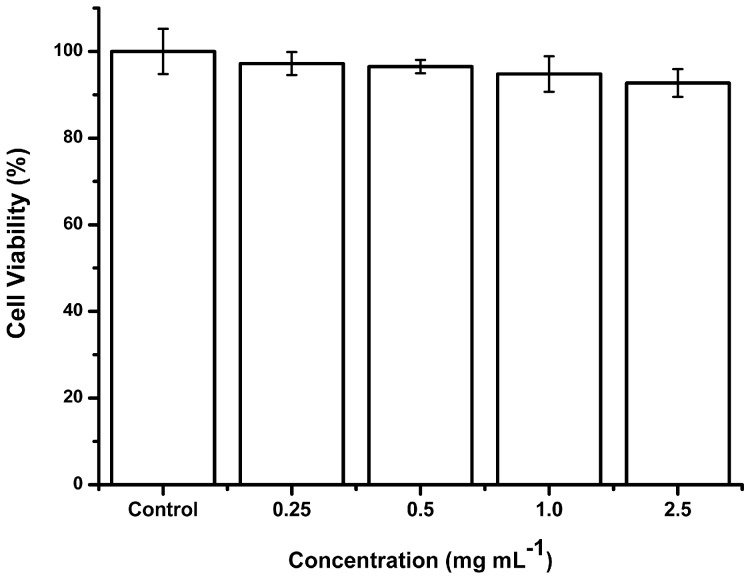
293T cells were cultured overnight for adherence and then treated with prepared ethosomal gels at different concentrations from 0.25–2.5 mg·mL^−1^ for 24 h.

As shown in Figure 1, these morphologies of 5-FU EGs appeared as spherical or oval, and have multilamellar structure observed by TEM. These lamellas coating the core of 5-FU EGs may result from the layer-by-layer synthesis route of EGs (Figure 1B). The average size of freshly prepared 5-FU EGs was 132.37 ± 32.17 nm (*n* = 216). These data correlate well with the vesicular size observed by the TEM images. In addition, the cytotoxicity of the ethosomal gels was examined by using a standard methyl thiazoly tetrazolium (MTT) assay* in vitro*. Normal cells line 293T cells were first cultured overnight for adherence and then treated with ethosomal gels at various concentrations from 0.25–2.5 mg·mL^−1^ for 24 h. The cells without treatment of ethosomal gels were used as control. It was found that a low cytotoxicity was induced by ethosomal gels in 293T cells in the test concentration range after 24 h incubation (shown in Figure 2).

### 2.2. The Size Stability of 5-FU Ethosomal Gels Stored at 4 ± 1 and 25 ± 1 °C

**Figure 3:**
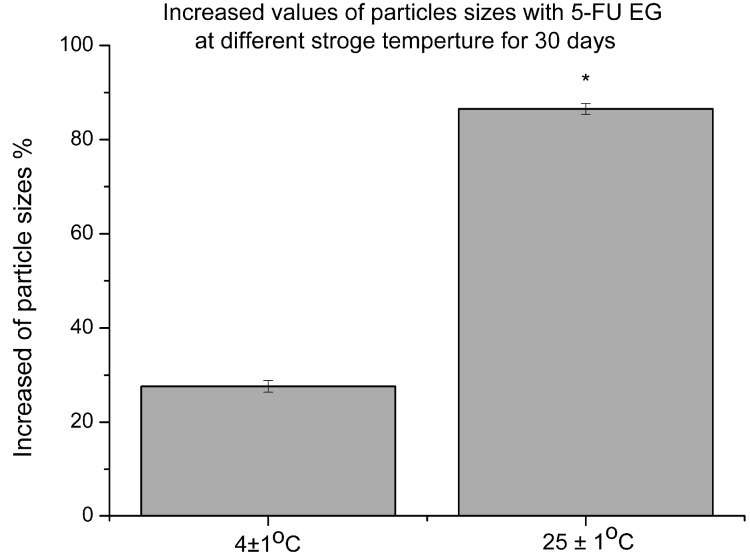
The increased values of particle size with liposomal gel loaded 5-FU at different storage temperatures. Values were expressed as the mean ± SD, using the independent-samples *t*-test. (* *p* < 0.05 compared to 5-FU EGs at 4 ± 1 °C for 30 days).

The stability of 5-FU EGs at different temperatures and times was further evaluated because of their fusion property. As shown in TEM images, some EGs remained stable at 4 ± 1 °C after 30 days (Figure 1C), while part of EGs tended to show complete fusion and aggregation at 25 ± 1 °C after a 30-day storage period (Figure 1D). By comparing the particle sizes of freshly prepared 5-FU EGs with those after 30 days of storage (Table 1), the average value of particles’ sizes increased from 132.37 ± 32.17 nm to either 167.29 ± 41.03 nm at 4 ± 1 °C or 242.1 ± 43.86 nm at 25 ± 1 °C, respectively. The maximum increase of the particle size was 34% ± 0.52% and 109% ± 0.46% at 4 ± 1 °C, at 25 ± 1 °C, respectively (Table 1 and Figure 3). Both absolute increase and the variation show a significantly better physical stability at 4 ± 1 °C than at 25 ± 1 °C. The results of the size for 30 days of storage showed that 5-FU EGs had a better stability in 4 ± 1 °C than that in 25 ± 1 °C. Gel formulation has a good biocompatibility that makes it closely adhere to function and penetrate the skin, and mucosa to obtain drug efficacy. At the same time, a gel formulation swells by absorbing water to form a hydration gel layer and is widely used in drug controlled release systems [24]. In this study, we prepared a 5-FU ethosome suspension with a carbomer hydrogel matrix to obtain 5-FU EGs which keeps it functional, maintains the stability of particle size for a long time and reduces the liposome suspension vesicle aggregation. These data have shown that 5-FU EGs storage at 4 ± 1 °C after 30 days still has good size stability (Figure 1 and Figure 2 and Table 1).

**Table 1:** Values of ethosome sizes loaded with 5-FU remained stable at different storage temperature for 30 days (nm) (*n* = 16).

Group	Original Size	Stored at 4 ± 1 °C	Stored at 25 ± 1 °C
5-FU EGs	132.3 ± 32.17 nm	167.29 ± 41.03 nm	242.09 ± 43.86 nm (*)

5-FU EGs: 5-FU Ethosomal Gels; * *p* < 0.05 compared to 5-FU EGs original size by using one-way ANOVA.

### 2.3. Ethosomal Gels Labeled with Rhodamine 6G Has the Ability to Penetrate Rabbit Ear Hypertrophic Scars

**Figure 4:**
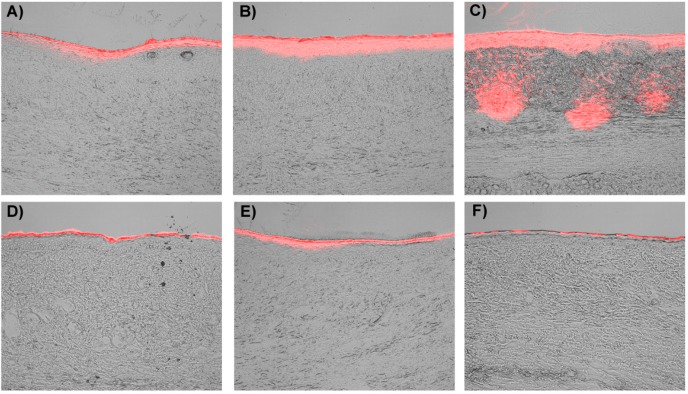
EGs labeled red fluorescence (Rhodamine) penetrated the scar tissues after (**A**) 1 h; (**B**) 6 h; (**C**) 24 h. PBS Gel labeled red fluorescence (Rhodamine) penetrated the scar tissues after (**D**) 1 h; (**E**) 6 h; (**F**) 24 h. (Original magnification of **A**–**F** is 20×).

**Figure 5:**
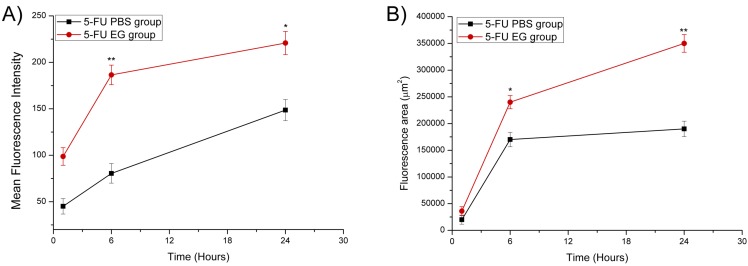
Fluorescence analysis in rabbit scar tissues following application of either ethosomes gel or phosphate buffer saline (PBS) gel containing Rhodamine 6G for 1, 6 and 24 h. Analysis was conducted using Release Version 4.0 SP2 image analysis software (Analytik Jena, Thuringia, Germany). (**A**) Mean fluorescence intensity of EGs* vs.* PBS gels; (**B**) Fluorescent areas of EGs *vs.* PBS gels. All time points represent the mean ± SD (*n* = 6). * represents* p* < 0.05 and ** represents* p* < 0.01.

Figure 4 shows the penetration efficiency of EGs labeled with Rhodamine 6G in rabbit ear hypertrophic scars. These images of hypertrophic scar cross-sections are perpendicular to the scar surface. Fluorescence intensities indicate that EGs penetration is a time-dependent process. After 1 h, it can be seen that both EG groups and the phosphate buffer saline (PBS) gel group assemble over the superficial epidermis of the hypertrophic scar. The fluorescence permeation in the EGs group was similar to that in PBS gel group (Figure 4A,D). However, the area of fluorescence penetration was much larger in the EGs group than that of the PBS gel group after treatment of 6 h (Figure 4B,E). After application with EGs for 24 h, the fluorescence penetrated massively in a scattered way and occupied partially the deep dermis of the hypertrophic scar. After application of PBS gel for 24 h, the fluorescence penetrated only to the superficial epidermis (Figure 4C,F). Additionally, the fluorescence intensity of EGs group was much greater than that of the PBS gel group after application for 6 and 24 h (Figure 5A). Similarly, compared to the PBS group, the fluorescence of EGs group also occupied a larger area after application for 6 and 24 h (Figure 5B). Especially after application for 24 h, the drug penetrated the stratum corneum and vital epidermis by using EGs, while delivering only to the superficial stratum corneum of skin without using EGs.

Although, the rheological synergy study suggests that as soon as mucus and mucoadhesive penetrate, EGs are likely to interact and form a surface gel layer that will substantially inhibit any further interpenetration. In this study, EGs were fully evaluated for rabbit ear hypertrophic scar, by assessing the penetration efficiency of EGs labeled with Rhodamine 6G in different periods (Figure 4 and Figure 5). The results showed that EGs had scattered to a large area in the dermis from the superficial epidermis after treatment of 24 h. After treating for 24 h, the fluorescence penetrated massively in a scattered fashion and occupied the deep dermis of the hypertrophic scar partially. All of these phenomena may relate to the carbomers matrix of EGs: It is the composition of 5-FU EGs which has been adapted to interact with mucoadhesive materials and a mucous membrane. At the same time, there also is another interesting phenomenon: the fluorescence which is Rho EGs lasted for 24 h penetrated in a massively scattered fashion different to the dispersion pattern. It may relate to there being a surface gel layer between the mucoadhesive and mucous membrane, poor liquidity ability of colloidal solution and the EGs’ vesicle crosslinking with thick carbomer colloidal solution. Even so, it showed EGs can also permeate epidermis into the dermal tissue of rabbit hypertrophic scars.

### 2.4. 5-FU Ethosomal Gels Have a Positive Effect on Hypertrophic Scar Reduction

**Figure 6:**
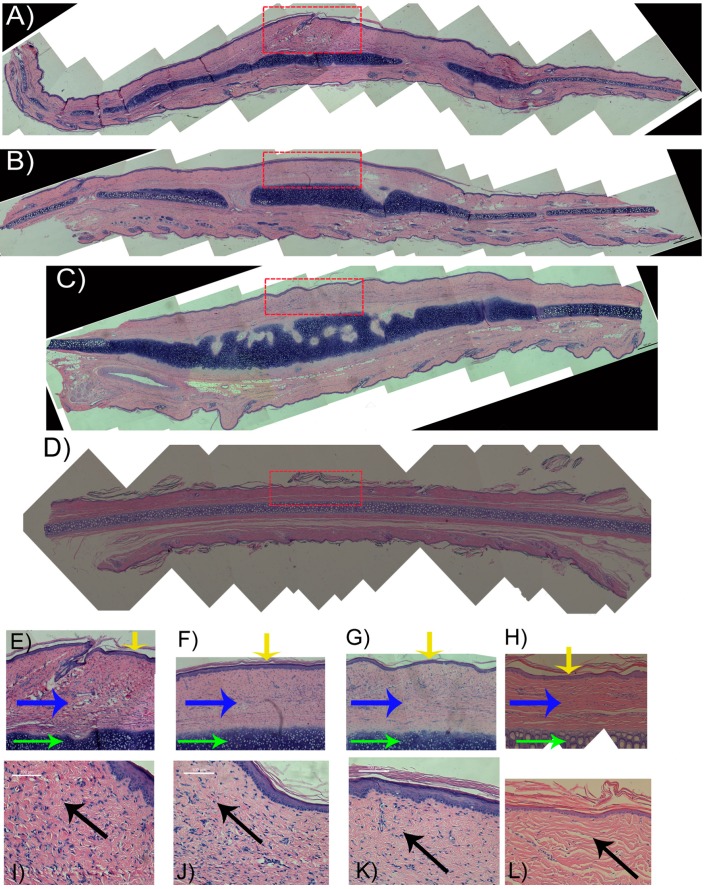
Haematoxylin and Eosin (H&E) histological analysis of scars harvested after application of different treatments for 30 days. (**A**,**E**,**I**): Control group; (**B**,**F**,**J**): 5-FU EGs group; (**C**,**G**,**K**): 5-FU Gels group; (**D**,**H**,**L**): Normal skin; (**A**–**D**) over-view of H&E images in different groups. Representative images labeled by red dot lines are shown in details in (**E**–**H**), respectively. Dermal thickness was significantly higher in control group scars (**E**) than in 5-FU Gels group (**G**) and 5-FU EGs group (**F**). Representative histological (**I**–**L**) observations were made in the hypertrophic scar tissue. (Original magnification, 2× for (**A**–**D**); 20× for (**E**–**H**), 200× for (**I**–**L**)). The yellow arrow labeled epidermis; the blue arrow showed dermis (scar tissues); the green was cartilage tissue and the black arrow showed collagen fibers.

**Figure 7:**
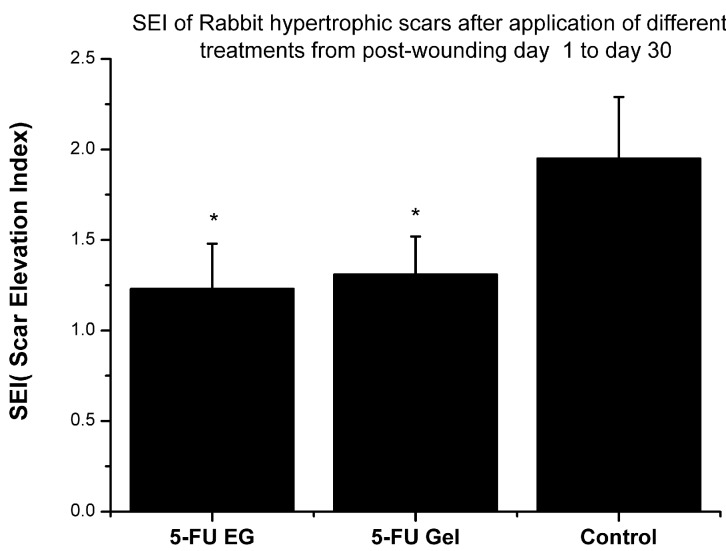
Scar elevation index (SEI) of rabbit hypertrophic scars after application of different treatments from postwounding day 1 to day 30. Values were expressed as the mean ± SD. *n* = 14*.* * *p* < 0.05 compared to control samples using one-way ANOVA.

Figure 6 shows hypertrophic scar (H&E) morphological profiles of control group (Figure 6A,E,I) (not using any treatment), using 5-FU EGs(Figure 6B,F,J), 5-FU gels(Figure 6C,G,K) and normal skin(Figure 6D,H,L). In H&E stained tissues, the profiles contain a large number of collagen fibers with crisscross, spiral, and circular patterns and show a significant amount of disorderly arranged fibroblasts (Figure 6A–C).The profiles of dermal thickness in both the 5-FU EGs group and 5-FU gels group are thinner than the profile in the control group (Figure 6E–G). As shown in Figure 6B,C, regenerative cartilage tissue in the 5-FU EGs group and 5-FU gels group is also observed. By comparing the Scar Elevation Index (SEI), dermal thickness was significantly higher in control group scars than in others treated scars with *p* < 0.05 (Figure 6 and Figure 7). In addition, there is no significant difference in the value of SEI between application of 5-FU EGs and 5-FU gel groups (*p* > 0.05).

The results showed that for the 5-FU EGs and 5-FU gels group, the profiles of dermal thickness were thinner than in the control group with no treatment. In addition, the collagen I was involved in the process of fibro proliferative scarring [25]. Compared to the other two groups, there is a larger number of collagen fibers and a much more significant amount of fibroblasts, relatively orderly arranged, in 5-FU EGs. In the 5-FU EGs treatment groups, the levels of collagen I decreased dramatically after 30 days of treatment with 5-FU gel using western blots. These data suggested that hypertrophic scars in rabbit ear had been relieved and secretion collagen I was inhibited by using 5-FU EGs.

### 2.5. 5-FU Ethosomal Gels Reduces Collagen I Expression in Hypertrophic Scar Tissue

**Figure 8:**
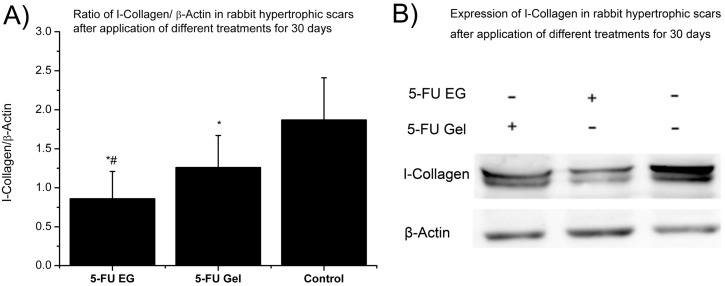
(**A**) Expression of I-Collagen in rabbit hypertrophic scars after application of different treatments for 30 days; (**B**) Ratio of I-Collagen/β-Actin * *p* < 0.05 compared to control samples; # *p* < 0.05 compared to 5-FU Gel.

Expression of collagen I in the scar tissue can reflect the hyperplasia in the scar tissue. Compared to 5-FU gel group and control group, the 5-FU EGs group had the lowest level of collagen I after 30 days treatment (Figure 8). There is a significant difference between 5-FU EGs group and 5-FU gels group for secretion of collagens I (*p* < 0.05). This was also the case between the 5-FU EGs group and control group (*p* < 0.05). The collagen I in rabbit ear hypertrophic scar was up-regulated after application of 5-FU EGs for 30 days.

## 3. Experimental Section

### 3.1. Materials

Soybean phosphatidylcholine (Phospholipon 90), absolute alcohol, 5-FU, carbomers, propanetriol, triethanolamine and Rhodamine 6G were purchased from Sigma, Shanghai, China, and all chemicals were used as received without further purification.

### 3.2. Preparation of 5-FU Ethosomal Gels (5-FU EGs)

Ethosomal suspensions were prepared as described by Touitou* et al.* [8]. Briefly, EGs were composed of 2% (*w*/*v*) soybean phosphatidylcholine (PC), 30% (*v*/*v*) absolute alcohol and 0.8% (*w*/*v*) 5-FU and Phosphate Buffered Solution (PBS, pH 7.4) to 100% (*w*/*v*). The PC was dissolved in ethanol. PBS containing 5-FU was added slowly in 70 µL/s with constant mixing at 700 rpm in a sealed container using magnetic stirring apparatus (IKA RH-control, Staufen, Germany) at 30 °C. Mixing was continued for an additional 30 min and then left to cool at room temperature. At room temperature, the mixture was put in an ultrasonic bath for 30 min to obtain the smaller particles. Ethosomal formulations were prepared by extruding the particles through 50 nm pore size polycarbonate membrane filters 30 times to produce the appropriated size of ethosomal suspensions with 5-FU, with an average diameter of less than 100 nm.

Then, 3.2% (*w*/*v*) carbomers as well as 8% (*w*/*v*) propanetriol were both added into the solution of double-distilled water. The resulting mixture was stirred for 2 h at room temperature and neutralized with triethanolamine to give a carbopol gel matrix with a pH value of 7.4. Ethosomal suspensions containing 0.8% (*w*/*v*) 5-FU were mixed slowly with carbopol gel matrix at a volume ratio of 1:1 with constant mixing at 500 rpm in a sealed container under magnetic stirring apparatus at room temperature. The resulting mixture was stirred for 2 h and the final concentration of 5-FU was 0.4% (*w*/*v*) in 5-FU EGs.

To prepare the 5-FU PBS gels with 0.4% (*w*/*v*) concentration, 0.8% (*w*/*v*) 5-FU was dissolved in Phosphate Buffered Solution (PBS, pH 7.4). Then, 0.8% (*w*/*v*) 5-FU PBS was mixed with carbopol gel matrix at a volume ratio of 1:1 with continuing stirring to form 5-FU PBS Gels. The final concentration of 5-FU was 0.4% (*w*/*v*). 5-FU Gels having the concentration of 0.4% (*w*/*v*) acted as a control group.

### 3.3. Preparation and Fluorescence Labeling of Ethosomes

Rhodamine 6G, the fluorescence labeling marker of ethosomal suspension was prepared with the same procedure as mentioned above for the 5-FU ethosomal suspensions. Ethosomal suspensions were composed of 2% (*w*/*v*) soybean phosphatidylcholine (PC), 30% (*v*/*v*) absolute alcohol, 0.06% (*w*/*v*) Rhodamine 6G, and Phosphate Buffered Solution (PBS, pH 7.4) to 100% (*w*/*v*). Ethosomal Rhodamine 6G formulations were prepared by extruding the particles through 50 nm pore size polycarbonate membrane filters 30 times to produce the ethosomal Rhodamine 6G suspensions.

Rhodamine 6G ethosome colloidal suspension was mixed with an equal volume of the carbopol gel with continuing stirring to form Rhodamine EGs. The final concentration of Rhodamine 6Gwas 0.03% (*w*/*v*).

To prepare the Rhodamine PBS Gels with 0.03% (*w*/*v*) concentration, 0.06% (*w*/*v*) Rhodamine 6G was dissolved in Phosphate Buffered Solution (PBS, pH 7.4). Then, 0.06% (*w*/*v*) Rhodamine 6G PBS was mixed with carbopol gel matrix at a volume ratio of 1:1 to form 0.03% (*w*/*v*) Rhodamine PBS Gels.

In order to evaluate the cytotoxicity of EG, a modified MTT (3-(4,5-dimethylthiazol-2-yl)-2,5-diphenyltetrazoliumbromide) test was used in this study. Briefly, after incubation in different concentration of EG for 24 h, Human Embryonic Kidney 293T cells were washed three times with culture medium, and then 100 μL MTT (5 mg/mL in PBS) was added. After 4 h incubation at 37 °C, the reaction solution was carefully removed from each well and 200 μL dimethyl sulfoxide was added. The plates were gently agitated until the formazan precipitate was dissolved, followed by measurement of optical density (OD) values by spectrophotometry at 490 nm with an ElX-800 Microelisa reader (Bio-Tek Inc., Omega, VT, USA).

### 3.4. Characterization of 5-FU Ethosomal Gels

5-FU EGs was diluted 10-fold with double-distilled water and stirred with constant 20 rpm for 2 h. Before Transmission Electron Microscopy (TEM) measurement, 20 µL samples were placed on the copper grid coated with carbon film and air-dried overnight. The samples were then prepared at room temperature by conventi-onal negative-staining methods using 3% phosphotungstic acid buffer (pH 6.0). Samples were viewed on a Philips TEM JEM2010 electron microscope (Philips, Tokyo, Japan), with an accelerating voltage of 100 kV. Furthermore, the particle size of the samples was determined by Photon Correlations Spectroscopy (PCS). Samples were re-diluted with PBS to reach a count rate of 250–350 kHz. The instrument parameters were set as follows: automatic choice of channel width, vesicle mode, number weighting, and automatic change from Gaussian distribution mode to multimodal mode (Nicomp distribution mode) if the value for χ^2^ exceeded 3.0. Each sample was evaluated three times and expressed as mean ± standard deviation (SD).

### 3.5. In Vitro Stability Evaluation

5-FU EGs was stored for 30 days at 25 °C thermostatically controlled and 4 °C in a refrigerator, respectively. Clarity and the particle size of the samples were determined after 30 days of storage to evaluate their stabilities by TEM and PCS using the Zeta potential/Particle sizer 380 zls. Samples were re-diluted with PBS to reach a count rate of 250–350 kHz and as well as was determined by PCS. For TEM measurement, 5-FU EGs was diluted 10-fold with double-distilled water and stirred with constant 20 rpm for 2 h and placed on the copper grid overnight. Samples were evaluated on a TEM with an accelerating voltage of 100 kV.

### 3.6. Rabbit Ear Hypertrophic Scar Model Construction

Hypertrophic scars were obtained using Morris’s Methods [26]. All procedures were approved by the Care and Use of Laboratory Animals Institutes of Shanghai, China. The ethics identification code is 2013109; Date of approval is from 1 January 2013 to 31 December 2013. The project identification code is SYXK (Hu) 2013-0050. The experiments were carried out with the approval of the Animal Experimentation Ethics Committee of School of Medicine, Shanghai Jiao Tong University. Eighteen young adult (2.0–2.5 kg) female New Zealand White rabbits (Shanghai Si-Lai-Ke Experimental Animal Co., Ltd., Shanghai, China), were acquired and single-housed in a regulated environment (22 ± 2 °C), with food and water provided ad libitum throughout the experiment.

The rabbits were anesthetized with intramuscular injection of ketamine (45 mg/kg) and xylazine (7 mg/kg) [26] and skin preparation entailed hair removal. Five 10 mm-diameter wounds were created down to bare cartilage on the ventral surface of each ear with removal of perichondrium. Each wound was 15 mm apart from the each other to prevent potential interaction between them along the experimental protocol. Wounds were covered with sterile gauze for 1 day. After recovery from anesthesia, the rabbits were returned to their cages. In total, there were 180 wounds created on 18 rabbits. Hypertrophic scars appeared on day 30. It was considered an adequate hyperplastic scar model based when the ratio, recorded using a vernier caliper on day 30, between the top of scar tissue and the surrounding thickness was over 1.5.

### 3.7. Grouping and Treatment

On postoperative day 30 and afterwards, as an adequate amount of rabbit hyperplastic scars had formed, the scars were assigned into two treatment groups including 5-FU EGs group and 5-FU gels group. These scars without any treatment were taken as control group. Each ear was assigned randomly (left or right) as the 5-FU EGs/5-FU PBS gel-treated or the control one. In treated wounds, 5-FU EGs/5-FU gels were administered once daily to the scar, thinly coated with 0.2 mg/day/scar (0.785 cm^2^) and with half an hour massage [6,27,28]. The scars in the control group did not undergo any treatment. The scars in the two treated groups and control group were all harvested on day 30 from the day of initial application.

### 3.8. Rhodamine Ethosomal Gels and Rhodamine Phosphate Buffered Solution (PBS) Gels

Rhodamine EGs was tested* vs.* Rhodamine PBS Gels. Each ear was assigned randomly (left or right) to be the Rho EGs or Rhodamine PBS Gels. It was applied once to the scar as a thin coat with 0.05 g/scar (0.785 cm^2^) and with half an hour massage. The scars were harvested separately at 1, 6 and 24 h after administration.

### 3.9. Tissue Preparation for in Vivo Rabbit Ear Hypertrophic Scars Permeation of Rhodamine 6G by Confocal Laser Scanning Microscopy

After 1, 6 and 24 h of administration of the Rho EGs or Rhodamine PBS gels, the scars were harvested and excised leaving a margin of surrounding unwounded tissue. Each scar was washed in PBS three times, embedded in optimal cutting temperature compound, serially cut in 10 μm longitudinal sections by freezing microtome, and immediately was observed under Laser Scanning Microscope (LSM 510 ZEISS, Leica, Germany) with a Fluar 10×/0.5 numerical aperture objective lens. Optical excitation was carried out with a 543 nm He-Ne laser, and fluorescence emission was detected above 560 nm for Rhodamine 6G. Image analysis was performed using Image-Pro Software Release Version 4.5 (Media Cybernestics Inc., Rockville, MD, USA).

### 3.10. Tissue Preparation for Histological/Western Blot Analysis

On the 30th day, the rabbits of each group were sacrificed and the scars harvested. Scars were excised leaving a margin of surrounding unwounded tissue and to prevent scar shrinkage were put in paraffin sections, the samples contained the rabbit ear full-thickness scar skin and the cartilage. Half of each scar was fixed in 4% neutral buffered formaldehyde, dehydrated, embedded in paraffin, serial 4 mm × 5 μm cross sections, and stained with haematoxylin and eosin (H&E). The remaining half was placed in liquid nitrogen before being preserved in the −80 °C freezer for Western blot analysis.

### 3.11. Hematoxylin & Eosin Histological Examination of Scar Elevation Index (SEI)

Epidermis, dermis, perichondrium and cartilage thicknesses were measured on hypertrophic control scars and treated scars. The most objective criterion evaluated was the SEI [29,30]. SEI is defined as the ratio of height difference between maximal scar height and normal tissue height to the height of normal tissue surrounding the hypertrophic scars. An index value of 1 indicates that the wound healed essentially flat, with no scar hypertrophy. An index value of 1.5 indicates that the scar thickness or hypertrophy was increased 50% with reference to the non-scarred dermal thickness, and an index of 2 indicates a wound that healed with a 100% increased thickness compared with the normal dermal thickness. The SEI was measured twice by an examiner blinded to its measurements.

### 3.12. Western Blot Determination of Collagen I

Scar samples were cut to grains measuring 1 mm × 1 mm. The grains were harvested by adding into the culture Radio-Immunoprecipitation Assay (RIPA) (Sigma, Santa Cruz, CA, USA, P0278) and Proteinase inhibitor cocktail (Sigma, Santa Cruz, CA, USA, P8340) with a homogenizer at 20,000 r/min. After protein quantification, equal amounts of protein were heated at 100 °C for 5 min in a sample buffer, separated by 12% SDS–PAGE, and transferred to a polyvinylidenedifluoride membrane. After blocking, the membrane was incubated with Monoclonal Anti-Collagen Type I antibody (1:2000, Sigma, Santa Cruz, CA, USA) and Monoclonal Anti-β-Actin-Peroxidase antibody (1:25,000, Sigma, Santa Cruz, CA, USA). Immunoreactive proteins (110–120 kD for Collagen I and 42 kD for β-Actin) were visualized by enhanced chemiluminescence after incubation with HRP-conjugated goat anti-mouse IgG (1:8000).

### 3.13. Data and Statistical Analysis

Continuous variables are reported as mean ± standard deviation (SD). An independent sample *t*-test or nonparametric independent sample test are adopted to analyze the differences between variables of both groups. Comparisons of scar elevation index among treatment and control groups were performed using a one-way ANOVA followed by Tukey *post hoc* analysis. In addition, densitometric assessment of the bands on the autoradiogram in Western Blot was performed using Image-Pro Software and was analyzed by one-way ANOVA followed by Tukey *post hoc* analysis. SPSS Version 18 (SPSS Inc., Chicago, IL, USA) was used for statistical analysis. A *p*-value of <0.05 was chosen to indicate statistical significance.

## 4. Conclusions

In this study, we successfully prepared 5-FU EGs and evaluated its stability* in vitro*. Our experiments have shown that 5-FU EGs, stored at 4 ± 1 °C, after 30 days still have good size stability. This provides a feasible condition for hypertrophic scar treatment in the rabbit ear model. Notably, EGs could efficiently penetrate the rabbit ear scar tissues labeling with Rhodamine and after loading 5-FU in the rabbit model. Obviously, 5-FU EGs cause a decrease of collagen I expression in scar tissues. Therefore, 5-FU EGs have an ability to inhibit hypertrophic scars. With the properties of durability and usability, the preparation and application of ethosomal gel formulation has been achieved and these research data have allowed us to establish ethosomal 5-fluorouracil gels that will be further investigated for their potential in reducing hypertrophic scars in humans, thereby making substantial progress in the field.
